# Oral potassium supplementation for management of essential hypertension: A meta-analysis of randomized controlled trials

**DOI:** 10.1371/journal.pone.0174967

**Published:** 2017-04-18

**Authors:** Jalal Poorolajal, Fatemeh Zeraati, Ali Reza Soltanian, Vida Sheikh, Elham Hooshmand, Akram Maleki

**Affiliations:** 1 Department of Epidemiology, School of Public Health, Hamadan University of Medical Sciences, Hamadan, Iran; 2 Research Center for Health Sciences, Hamadan University of Medical Sciences, Hamadan, Iran; 3 Noncommunicable Diseases Research Center, Hamadan University of Medical Sciences, Hamadan, Iran; 4 Medicinal Plants and Natural Products Research Center, Hamadan University of Medical Sciences, Hamadan, Iran; 5 Department of Biostatistics, School of Public Health, Hamadan University of Medical Sciences, Hamadan, Iran; 6 Department of Internal Medicine, School of Medicine, Hamadan University of Medical Sciences, Hamadan, Iran; 7 Department of Public Health, Neyshabur University of Medical Sciences, Neyshabur, Iran; Shanghai Institute of Hypertension, CHINA

## Abstract

**Importance:**

Increased dietary potassium intake is thought to be associated with low blood pressure (BP). Whether potassium supplementation may be used as an antihypertensive agent is a question that should be answered.

**Objective:**

To assess the effect of oral potassium supplementation on blood pressure in patients with primary hypertension.

**Search methods:**

We searched Medline, Web of Science, Scopus, Cochrane Central Register of Controlled Trials until October 2016. We also screened reference lists of articles and previous reviews. We applied no language restrictions.

**Selection criteria:**

We included randomized placebo-controlled clinical trials addressing the effect of potassium supplementation on primary hypertension for a minimum of 4 weeks.

**Data collection and analysis:**

We extracted data on systolic and diastolic BP (SBP and DBP) at the final follow-up. We explored the heterogeneity across studies using Cochran's test and I^2^ statistic and assessed the probability of publication bias using Begg's and Egger's tests. We reported the mean difference (MD) of SBP and DBP in a random-effects model.

**Results:**

We found a total of 9059 articles and included 23 trials with 1213 participants. Compared to placebo, potassium supplementation resulted in modest but significant reductions in both SBP (MD -4.25 mmHg; 95% CI: -5.96 to -2.53; I^2^ = 41%) and DBP (MD -2.53 mmHg; 95% CI: -4.05 to -1.02; I^2^ = 65%). According to the change-score analysis, based on 8 out of 23 trials, compared to baseline, the mean changes in SBP (MD -8.89 mmHg; 95% CI: -13.67 to -4.11) and DBP (MD -6.42 mmHg; 95% CI: -10.99 to -1.84) was significantly higher in the intervention group than the control group.

**Conclusions:**

Our findings indicated that potassium supplementation is a safe medication with no important adverse effects that has a modest but significant impact BP and may be recommended as an adjuvant antihypertensive agent for patients with essential hypertension.

## Introduction

Evidence has shown that high potassium intake can reduce blood pressure (BP), decrease the risk of developing cardiovascular disease, and mitigate the adverse effects of salt on blood pressure [1]. The World Health Organization (WHO) recommends a potassium intake of at least 90 mmol/day (3.5 g/day) from food for adults to reduce BP and risk of cardiovascular disease, cerebrovascular events, and coronary heart disease. Current evidence has shown no significant difference between the flavor and taste of potassium-enriched salt and regular salt [2]. The WHO also recommends a potassium intake of at least 90 mmol/day from food for children to control BP [3]. However, there is no need to give a supplement or specially formulated products because most people can replace needed potassium through food consumption [4,5].

Using potassium supplementation as an antihypertensive agent is a question that should be answered. Clinical trials to date have reported conflicting results on the BP-lowering effect of potassium supplementation. One previous meta-analysis conducted in 1997 demonstrated that potassium supplementation was associated with a remarkable reduction in mean systolic and diastolic BP (SBP and DBP) in people with or without hypertension. The authors suggested potassium intake for prevention and treatment of raised blood pressure, particularly in people who are not able to reduce their intake of sodium [6]. A systematic review was conducted in 1999 to provide evidence-based recommendations on dietary consumption and supplementation of potassium in the prevention and treatment of hypertension. The authors concluded that potassium supplementation above the recommended daily dietary intake should not be recommended as a treatment for hypertension [5]. A Cochrane meta-analysis, performed in 2006, reported no effect of potassium supplementation on primary hypertension. The authors recommended further investigation based on evidence from high quality long-term randomized controlled trials (RCTs) to explore whether potassium supplementation may reduce blood pressure and improve health outcomes [7]. Another meta-analysis, conducted in 2013, including RCTs and cohort studies, reported that increased potassium intake can reduce blood pressure in people with or without hypertension without side effect on blood lipid and catecholamine concentrations, or renal function. The authors suggested high dietary potassium intakes to prevent and control hypertension and stroke [8].

In this meta-analysis we just focused on the effect of potassium supplementation versus placebo on blood pressure in patients with essential hypertension. Furthermore, we planned to explore the dose-response relationship between potassium intake and blood pressure. We also displayed the temporal trends of evidence and how the conclusion may shift over a period of time. Therefore, we performed this updated meta-analysis to summarize the evidence from current randomized controlled trials to explore the benefits and harms of potassium intake for patients with essential hypertension and provide recommendations on the consumption of potassium supplementation as an adjuvant agent for management of hypertension.

## Methods

The Vice-chancellor of Research and Technology, Hamadan University of Medical Sciences, approved and funded this review. We wrote the report based on the PRISMA checklist of items for reporting systematic reviews and meta-analyses [9]. The supporting PRISMA checklist of this review is available as supporting information; see S1 PRISMA Checklist.

### Eligibility criteria

We included randomized controlled trials that reported the effect of potassium supplementation on SBP and DBP among patients with essential hypertension (SBP ≥140 mmHg and DBP ≥90 mmHg) [10]. We considered a minimum of 4 weeks of therapy to ensure that the intervention had sufficient time to produce an effect. Having a placebo group was necessary for inclusion in the review. Placebo included inert materials such as cellulose. We excluded trials that used potassium-enriched salts or potassium supplementation in combination with other minerals such as calcium or magnesium. We also excluded trials that assessed the prophylactic antihypertensive effect of potassium supplementation in normotensive people. The main outcome of interest was the variation in measured SBP and DBP readings at the final follow up.

### Information sources and search

We searched PubMed, Web of Science, Scopus, and the Cochrane Central Register of Controlled Trials until December 2015. We also searched the reference lists of included trials and previous relevant reviews. We applied no language limitations. We included the following search terms: (hypertension or hypertensive or blood pressure) and (potassium) and (clinical trial or controlled trial).

### Study selection

We pooled search results using EndNote software and removed duplicate records of the same report. Two of us (AM and EH) independently screened titles and abstracts and excluded the ineligible studies. Any disagreements were resolved through consensus. We retrieved and evaluated the full text of the potentially eligible trials for further evaluation. In cases where we found multiple reports of the same trail, we used the latest report.

### Data extraction

Two of us (AM and EH) independently extracted data from all included studies using an electronic datasheet prepared in Stata software. Disagreements were resolved by consensus. We extracted the following data from the eligible trials: first author’s name, year of publication, country, language, sex, age, study design (parallel, cross-over), sample size, dose (mmol/day), and mean (SD) SBP and DBP.

### Methodological quality

We assessed the methodological quality of the included studies using the Delphi checklist [11]. The checklist includes a set of items as follows. (1) Was a standard randomization performed? (2) Was the allocation of intervention concealed? (3) Was the patient blinded? (4) Was the care provider blinded? (5) Was the outcome assessor blinded? (6) Were the two groups similar at baseline? (7) Were the eligibility criteria well-defined? (8) Was the variability of the outcome presented? (9) Was an intention-to-treat analysis performed? On the basis of this checklist, we allocated a maximum score of nine to each study.

### Heterogeneity and publication biases

We explored the statistical heterogeneity across studies by chi-squared (Chi^2^) test [12] and measured its quantity by the I^2^ statistic [13] at the 5% significance level (P<0.05). We assessed the between-study variance using tau-squared (Tau^2^) statistic [12]. We investigated the possibility of publication bias by the Egger's and Begg's tests [14,15] and Trim & Fill method [16].

### Summary measures

We performed a meta-analysis to obtain a summary measure of the mean difference of BP between the intervention (receiving potassium supplementation) and control (receiving placebo) groups at the final follow-up using a random-effects model [17]. For assessing the intervention effect, a negative valued denoted a reduction in BP among the intervention group compared with the placebo group. All statistical analyses were performed at a significance level of 0.05 using Stata software, version 11 (StataCorp, College Station, TX, USA) and Review Manager, version 5.3.5.

### Sensitivity analysis

We used sequential algorithm [18] to achieve the minimum final I^2^ below the desired 50% threshold. For this purpose, for 23 trails included in this meta-analysis, we performed 23 new meta-analyses, while one trial was excluded from the calculations each time. The trail that was responsible for the largest reduction in I^2^ was dropped and a new set of 23−1 trials was created. When two or more trials caused exactly the same reduction in I^2^ by their exclusion, we dropped the trial with the largest reduction in Cochran's test. We continued this process until I^2^ decreased below the desired pre-set threshold. In the last step, if there was a possibility that more than one omitted trial could result in I^2^ dropping below the desired threshold, we reported the minimum I^2^.

## Results

### Description of studies

**Fig 1:**
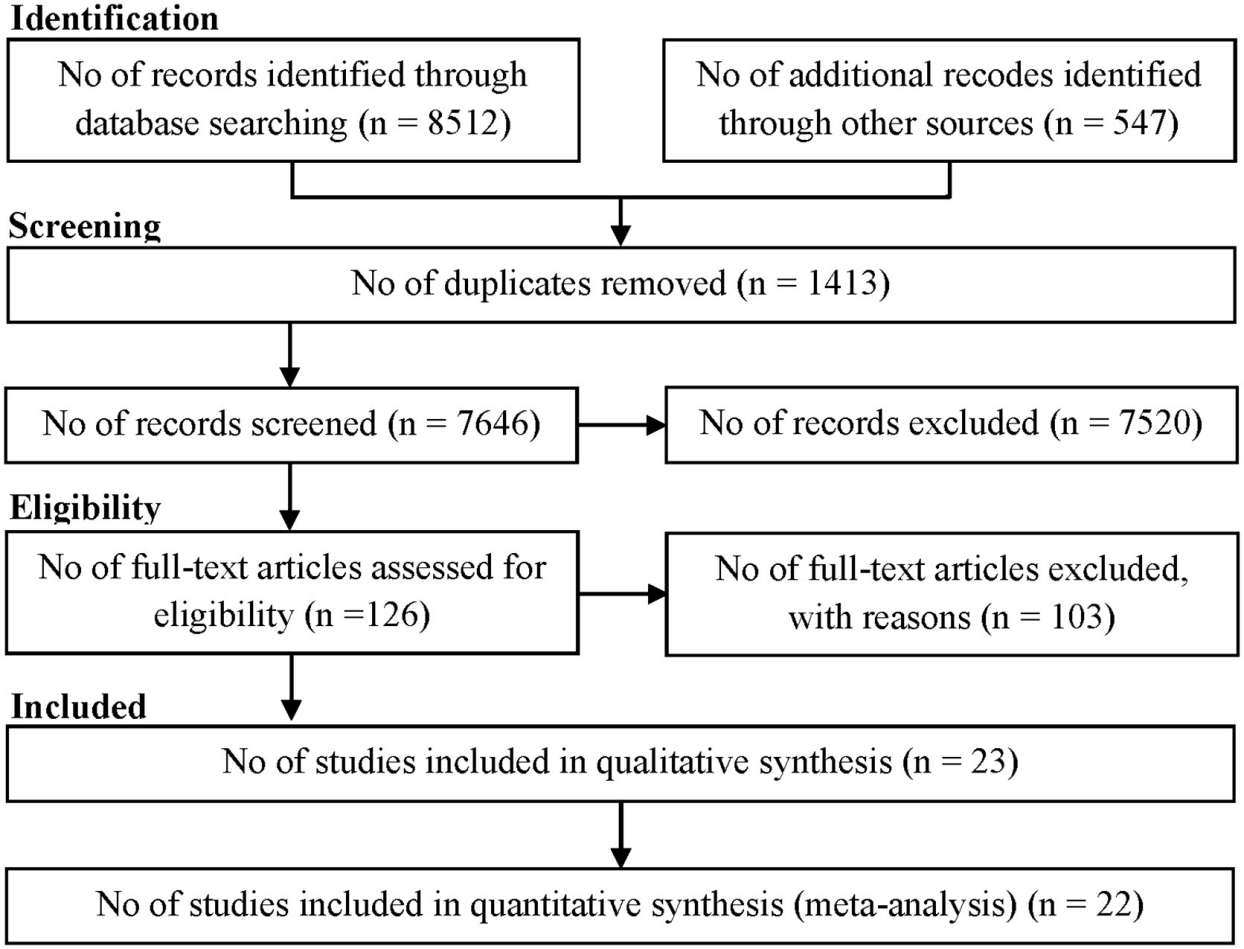
Flow of information through the different phases of the systematic review.

The results of the search process are shown in Fig 1. We found a total of 9059 trials, including 8512 articles through searching the electronic databases until October 2016 and 547 trials through screening reference list of the included trials. We excluded 1413 duplicates using EndNote software and 7520 ineligible trials through reading titles and abstracts. We also excluded 103 trials after checking the full-text reports, because they did not meet the inclusion criteria of this systematic review. Finally, 23 trials remained for meta-analysis, including 9 parallel and 14 crossover randomized placebo-controlled clinical trials involving 1213 participants [19–41]. All trials were published in English. The characteristics of the included trials are presented in Table 1.

**Table 1:** Summary of studies results.

First author, yr	Country	Mean age (yr)	Sex	Study design	Dose (mmol/d)	Sample size	Follow-up period (w)	Quality score ^a^
Forrester et al, 1988[19]	Jamaica	No data	Both	Parallel	48	46	No data	Abstract
Fotherby et al, 1992[20]	UK	75.0	Both	Crossover	60	18	4	6
Franzoni et al, 2005[21]	Italy	52.0	Both	Parallel	30	104	4	3
Gijsbers et al, 2015[41]	Netherlands	65.8	Both	Crossover	66	23	4	7
Grimm et al, 1988[22]	USA	58.0	Male	Parallel	96	312	12	5
Grobbee et al, 1987[23]	Netherlands	24.0	Both	Crossover	72	40	12	7
He et al, 2010[24]	UK	51.0	Both	Crossover	64	84	4	7
Heseltine et al, 1990[25]	UK	>65.0	Both	Crossover	60	10	4	7
Kaplan et al, 1985[26]	South Western	48.8	Both	Crossover	60	16	6	6
Kawano et al, 1998[27]	Japan	62.3	Both	Crossover	64	55	4	4
Lawton et al, 1990[28]	USA	24.0	Male	Crossover	100	10	4	4
MacGregor et al, 1982[29]	London	45.0	Both	Crossover	60	23	8	6
MacGregor et al, 1984[30]	England	45.0	Both	Crossover	64	23	4	7
Obel et al, 1989[31]	Kenia	40.0	Both	Parallel	64	48	16	7
Patki et al, 1990[32]	India	49.9	Both	Crossover	60	37	8	7
Rahimi et al, 2007[33]	Iran	48.8	Both	Parallel	102	56	4	3
Richards et al, 1984[34]	New Zealand	19–59	Both	Crossover	200	12	4	Abstract
Siani et al, 1987[36]	Italy	45.0	Both	Parallel	48	37	15	7
Siani et al, 1991[35]	Italy	30–65	Both	Parallel	30	47	52	6
Smith et al, 1985[37]	UK	53.0	Both	Crossover	64	20	4	4
Svetkey et al, 1987[38]	Singapore	51.0	Both	Parallel	40	101	8	8
Valdes et al, 1991[39]	Chile	50.0	Both	Crossover	64	24	4	7
Wu et al, 2006[40]	China	53.0	Both	Parallel	6	67	4	4

^a^ Assessment of methological quality of studies on the basis of Delphi checklist

### Main outcome measures

**Fig 2:**
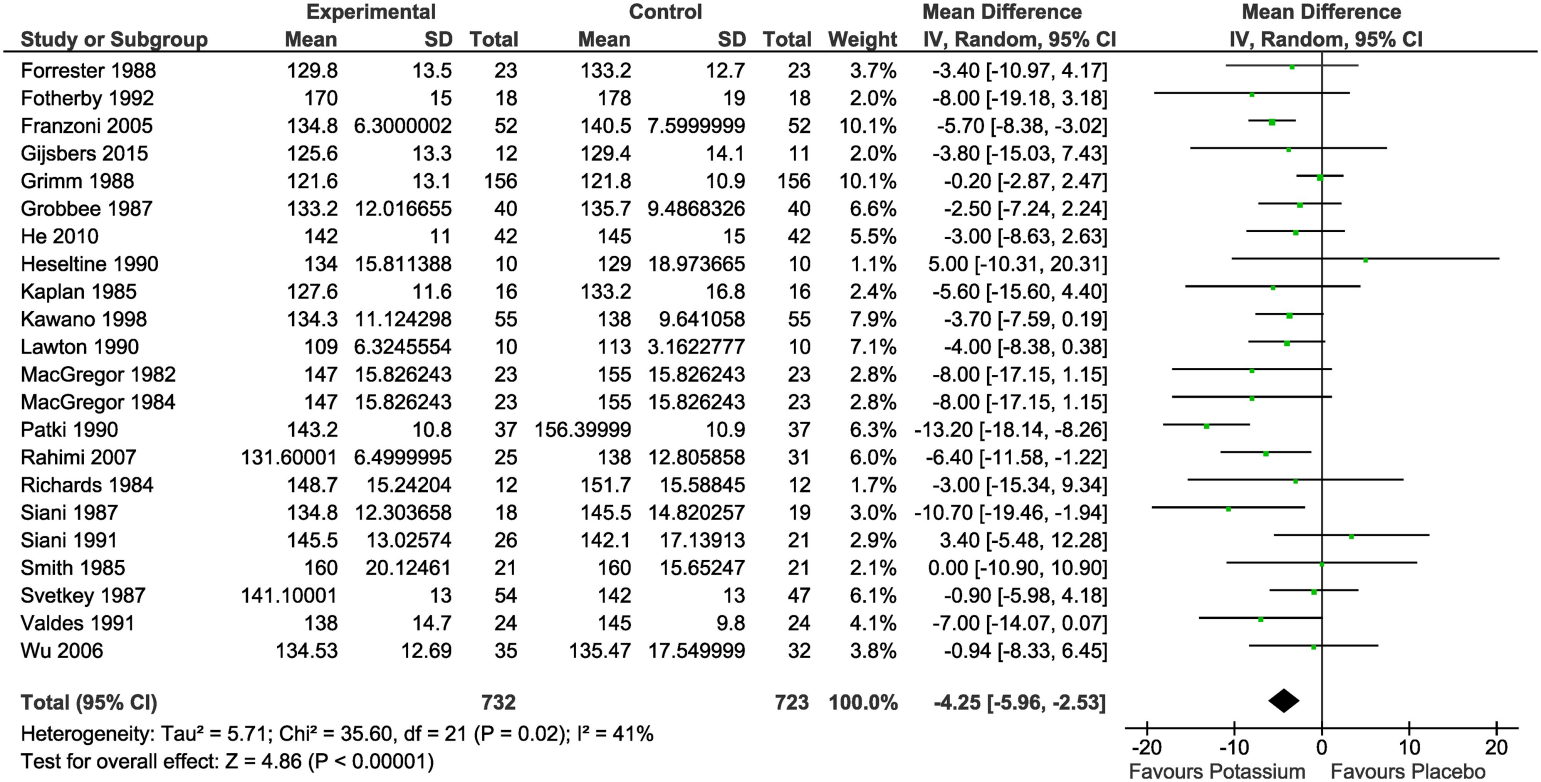
Meta-analysis of the randomized controlled trials reporting the effect of potassium supplementation on systolic blood pressure.

**Fig 3:**
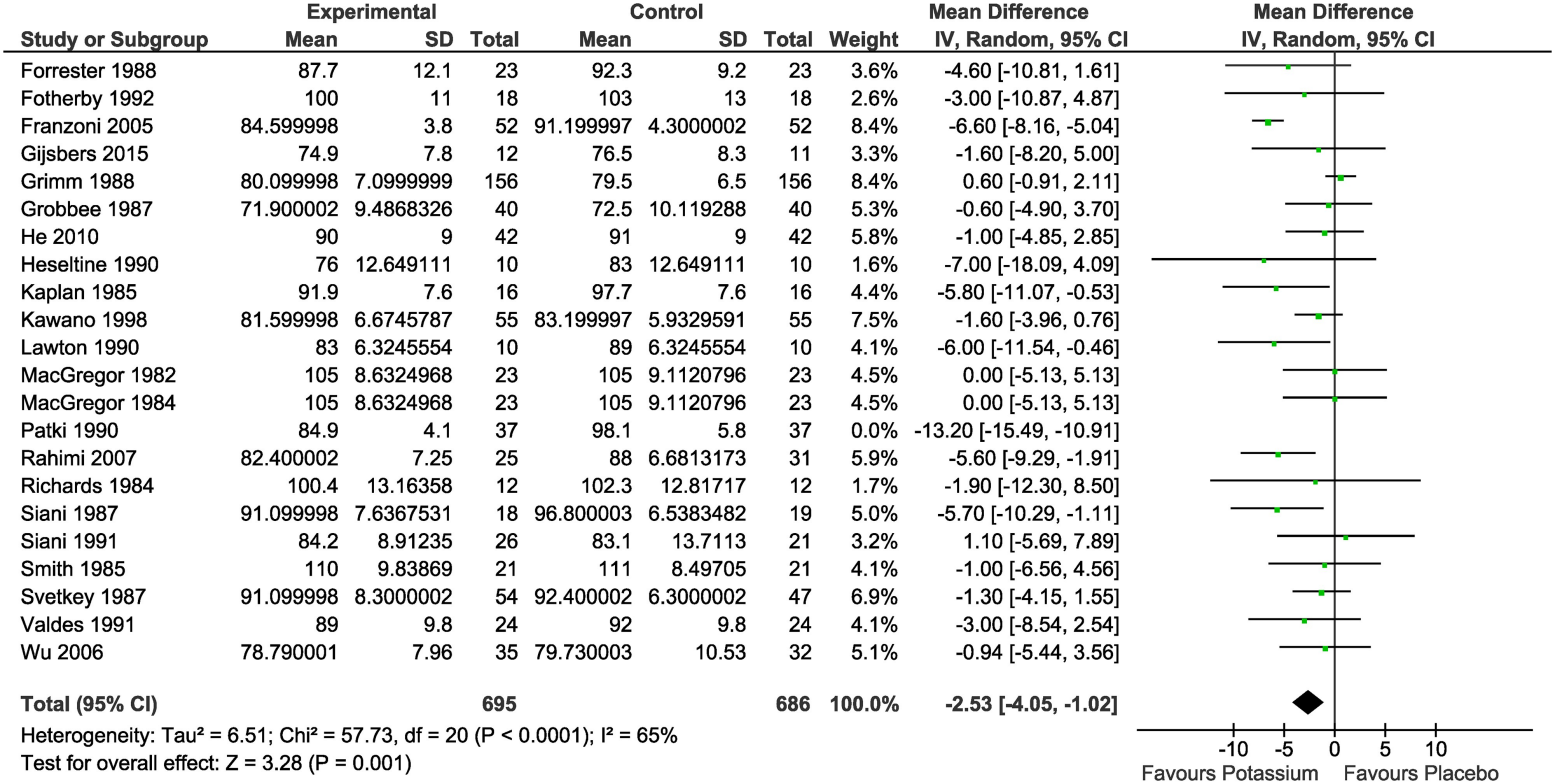
Meta-analysis of the randomized controlled trials reporting the effect of potassium supplementation on diastolic blood pressure.

Meta-analysis of the difference in mean BP between the intervention and control groups resulted in statistically significant reductions in both SBP (MD -4.25 mmHg; 95% CI: -5.96 to -2.53; I^2^ = 41%) (Fig 2) and DBP (MD -2.53 mmHg; 95% CI: -4.05 to -1.02; I^2^ = 65%) (Fig 3).

**Fig 4:**
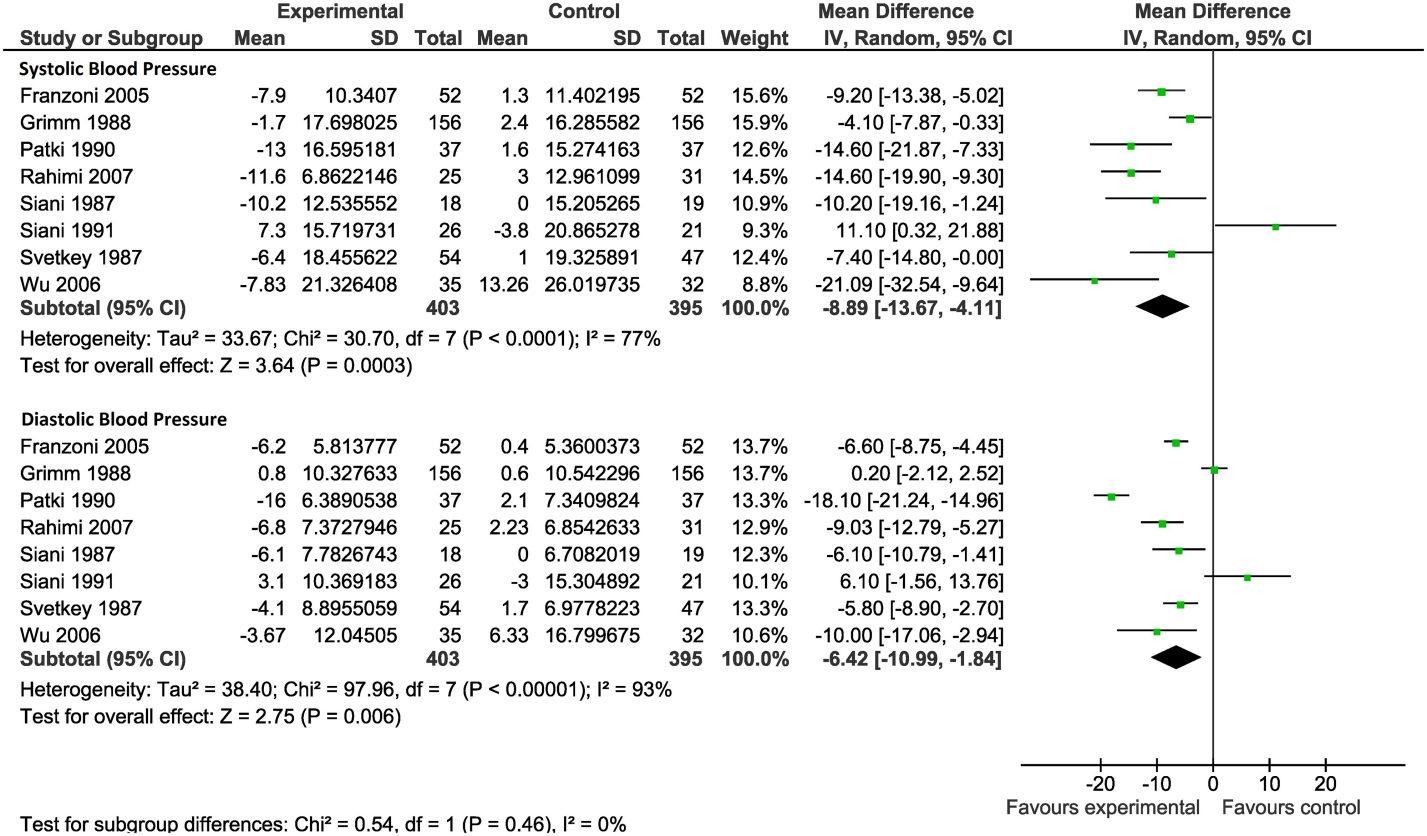
Meta-analysis of the randomized controlled trials reporting the mean change scores from baseline in systolic blood pressure (SBP) and diastolic blood pressure (DBP) in the intervention and control groups.

We performed change-score analysis based on 8 out of 23 trials that reported the changes in SBP and DPB in the two groups compared to baseline (Fig 4). As shown in this figure, compared to baseline, the mean changes in SBP (MD -8.89 mmHg; 95% CI: -13.67 to -4.11) and DBP (MD -6.42 mmHg; 95% CI: -10.99 to -1.84) was significantly higher in the intervention group than the control group.

There was an extreme value (outlier) among the included studies [31]. This trial consisted of 48 black patients with mild hypertension who received 64 mmol/day potassium supplementation for 16 weeks. According the results of this trial, potassium supplements resulted in significant decrease in mean SBP (MD -38 ±2.32 mmHg) and DBP (MD -18 ±1.15 mmHg) compared to control group. To establish homogeneity among the studies, we excluded this outlier from the meta-analysis.

Among the included studies, actually only two studies [22,38] including 428 patients, reported mild systemic adverse effect in a small number of patients as follows. Overall, among the patients who received potassium supplementation, two reported nausea or vomiting, 14 reported change in bowel habits (diarrhea, constipation), 15 reported abdominal pain, 12 reported gas (belching or flatulence), 2 reported headache, one reported anxiety, and one reported lethargy. Among the patients who received placebo, two reported nausea or vomiting, 15 reported change in bowel habits (diarrhea, constipation), 8 reported abdominal pain, 6 reported gas (belching or flatulence), 3 reported headache, one reported palpitation, one reported skin rash, one reported anxiety, one reported dizziness, and one reported lethargy.

### Publication bias

**Fig 5:**
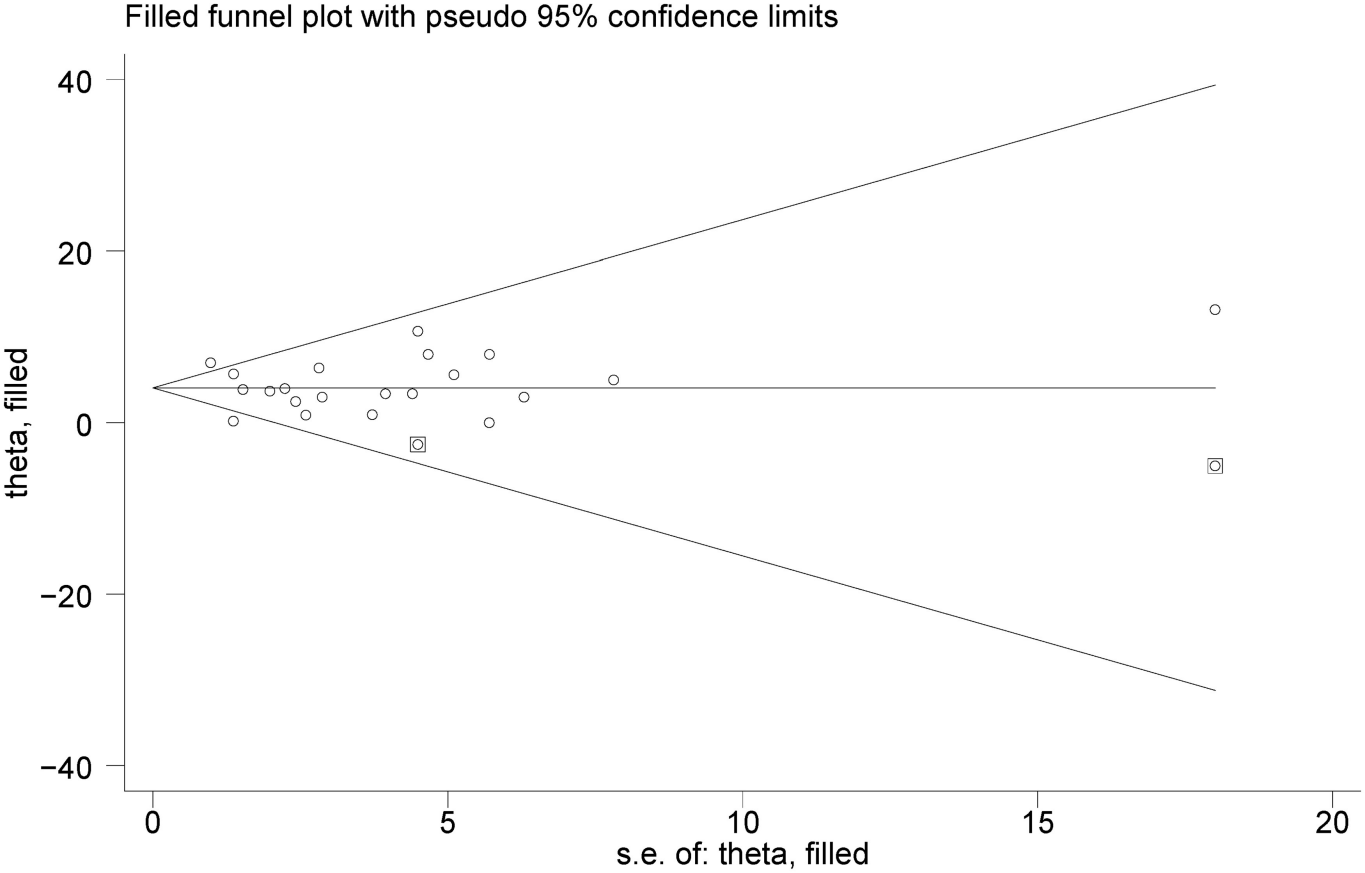
The filled funnel plot of the randomized controlled trials reporting the effect of potassium supplementation on systolic blood pressure.

We explored publication bias using Begg's and Egger's tests. According to these statistical tests, there was no evidence of significant publication bias among trials reporting the effect of potassium supplementation on SBP (P = 0.398 and P = 0.921) and DBP (P = 0.239 and P = 0.998), respectively. We also explored the possibility of publication bias using Trim and Fill method (Fig 5). This statistical method is a rank-based data augmentation technique that estimates the number and outcomes of missing studies and corrects the results of meta-analysis by incorporating the theoretical missing studies [21]. Based on this method, we found two potentially missing studies. However there was no statistically significant difference between the mean difference of SPB resulted from original meta-analysis -4.25 (95% CI: -5.96, -2.53) and the mean difference of SPB resulted from Trim and Fill method -4.07 (95% CI -5.32, -2.82). This method confirmed the absence of publication bias.

### Heterogeneity and sensitivity analysis

There was low statistical heterogeneity among trials reporting the effect of potassium supplementation on SBP (I^2^ = 41%) but there was evidence of moderate heterogeneity among trials addressing the effect of potassium intake on DBP (I^2^ = 65%). We performed a meta-regression to determine the causes of heterogeneity among results of studies (Table 2). We considered mean difference of SBP and DBP as the dependent variable and covariates such as potassium supplementation dosage, duration of follow-up, and participants' mean age as predictors. The result of meta-regression revealed that the association between potassium dosage, follow-up period and mean age were not statistically significant, therefore they did not play an important role in the heterogeneity across studies.

**Table 2:** Result of meta-regression analysis for exploring sources of heterogenity considerring systolic and diastolic blood pressure as the dependent variables.

Variables	Coefficeint	SE	t	*P* value	95% CI	
**Systolic blood pressure (mmHg)**						
Potassium dosage (mmol/day)	-0.04501	0.06096	-0.74	0.472	-0.17575	0.08572
Follow-up period (week)	-0.00348	0.02621	-0.13	0.896	-0.05969	0.05273
Age mean (year)	-0.39616	0.20750	-1.91	0.077	-0.84121	0.04890
Constant	9.31997	4.06457	2.29	0.038	0.60233	18.03760
**Diastolic blood pressure (mmHg)**						
Potassium dosage (mmol/day)	-0.05228	0.06440	-0.81	0.433	-0.19260	0.08804
Follow-up period (week)	-0.00028	0.02470	-0.01	0.991	-0.05410	0.05353
Age mean (year)	-0.16892	0.19584	-0.86	0.405	-0.59562	0.25777
Constant	6.42272	4.28737	1.50	0.160	-2.91866	15.76410

We also performed sensitivity analysis on the basis of the sequential algorithm to achieve between-study homogeneity. We achieved the minimum I^2^ below desired threshold (50%) by omitting two trials [21,32] from the meta-analysis (MD -1.83; 95% CI: -2.93 to -0.73; I^2^ = 22%).

### Subgroup analysis

**Fig 6:**
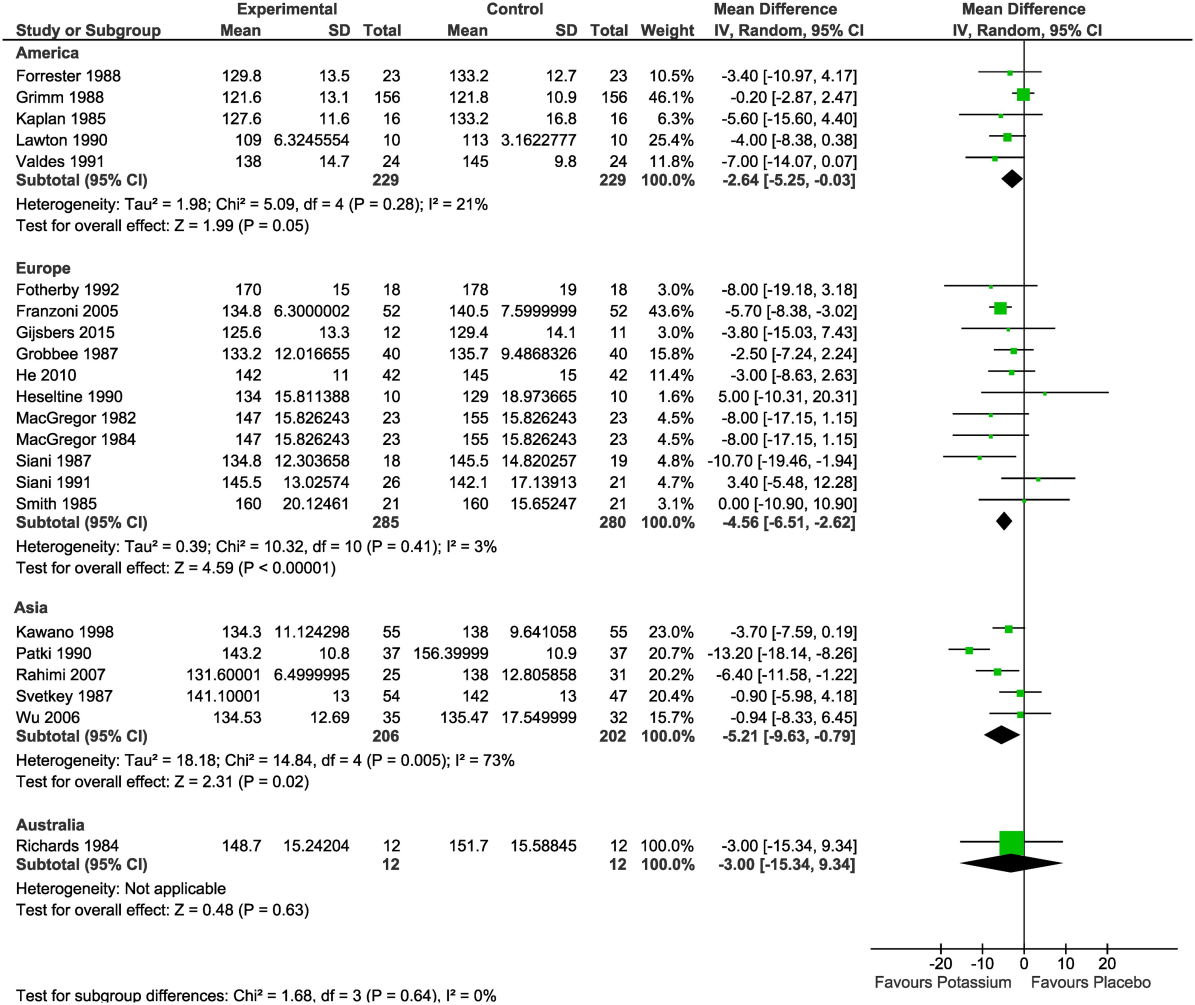
Meta-analysis of the randomized controlled trials reporting the effect of potassium supplementation on systolic blood pressure (SBP) by continent.

We conducted a subgroup analysis to explore the possibility of heterogeneity in the effect of potassium supplementation on SBP and DBP by continent. As shown in Fig 6, the mean difference in SBP was -2.64 (95% CI: -5.25 to -0.03) mmHg in America; -4.56 (95% CI: -6.51 to -2.62) mmHg in Europe; -5.21 (95% CI: -9.63 to -0.79) mmHg in Asia; and -3.00 (95% CI: -15.34 to 9.34) mmHg In Australia (based on one trial).

**Fig 7:**
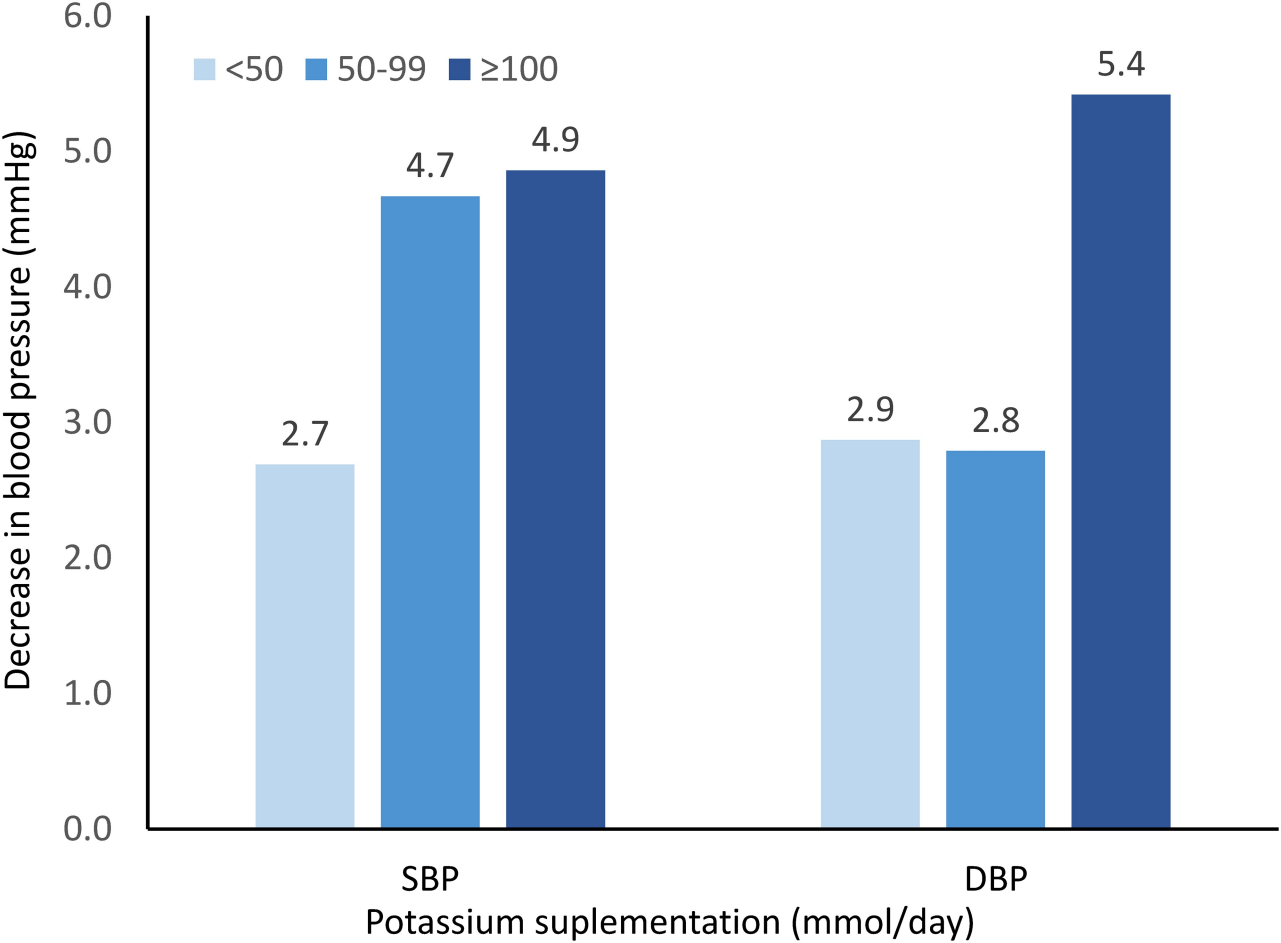
The dose-response relationship between potassium intake and reduction in systolic blood pressure (SBP) and diastolic blood pressure (DBP).

We conducted a subgroup analysis to explore the dose-response relationship between potassium intake and blood pressure. According to the potassium supplementation dosage, we divided the trials into low-dose (<50 mmol/day), moderate-dose (50–99 mmol/day), and high-dose (≥100 mmol/day) and then performed meta-analysis for each category. According to the results presented in Fig 7, there was a dose-response relationship between potassium intake and reduction in systolic and diastolic BP.

### Further analysis

**Fig 8:**
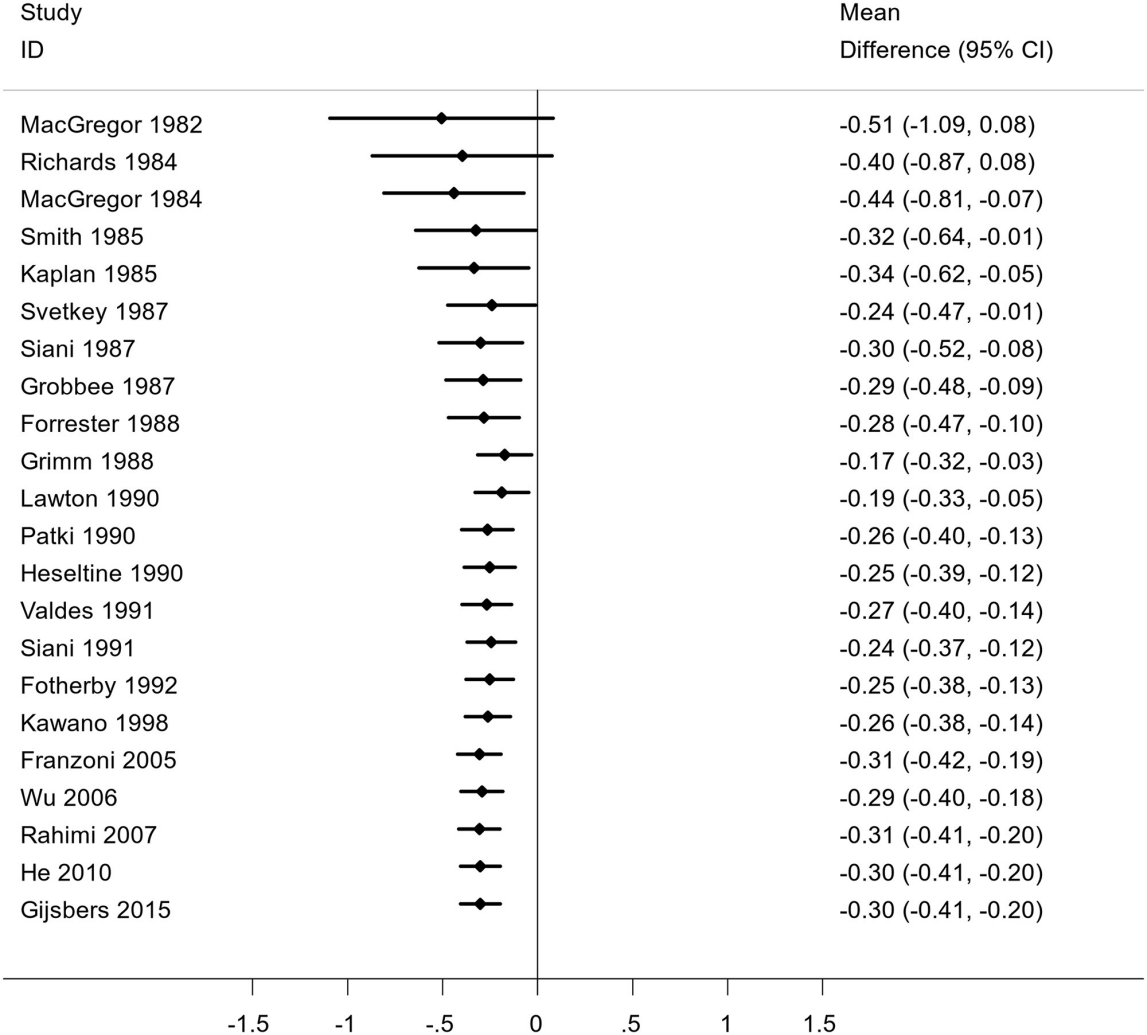
Cumulative meta-analysis of the randomized controlled trials reporting the effect of potassium supplementation on systolic blood pressure.

We sorted studies chronologically and performed a cumulative meta-analysis to detect temporal trends of evidence and to see how the evidence has shifted over time. A cumulative meta-analysis is a meta-analysis run first with one study, then repeated with a second study added, then a third, and so on. Accordingly, the first horizontal line indicates the effect based on one trial, the second line indicates the cumulative effect based on two trials, and so on. The results of cumulative meta-analysis is given in Fig 8. As we move down the plot, we see a consistency in the results of consecutive experiments and the effect size tends to stabilize. The presence of consistency between the results means that the results continually favor the treatment effect of potassium and thus no further experiment is required to make a conclusion about the impact of potassium supplementation on BP.

## Discussion

Our findings indicated that potassium supplementation had a statistically significant effect on both SBP and DBP. Subgroup analyses revealed an evidence of dose-response relationship between potassium intake and BP reduction. Accordingly, potassium supplementation had a clinically modest impact on essential hypertension and thus may be used as an adjuvant antihypertensive agent.

In addition to conventional comparing SBP and DBP in the intervention and control groups, we performed change-score analysis to explore the changes in SBP and DPB in the two groups compared to baseline. Based on this method, the mean differences between the intervention and control groups became more obvious. The reason was that change-score analysis with baseline adjustment provided a better estimate of the effect of potassium supplementation on blood pressure.

There was evidence of heterogeneity (low P value and a large Chi^2^ statistic) among the trials addressing the effect of potassium supplementation and DBP. However care should be taken in the interpretation of the Chi^2^ test, since it has low statistical power in the situation of a meta-analysis when studies have small sample size or are few in number. On the other hand, when there are many studies in a meta-analysis, the test has high power to detect a small amount of heterogeneity that may be clinically unimportant [42]. In such situation I^2^ statistic is useful method because this statistic has been developed for quantifying inconsistency across studies. It moves the focus away from testing whether heterogeneity is present to assessing its impact on the meta-analysis [13]. According to I^2^ statistic there was low to moderate heterogeneity across studies that may be due to clinical and methodological diversity among studies.

Potassium is an essential nutrient that plays a key role in maintenance of body fluid, acid-base balance, and normal cell structure and function [43]. There is a significant inverse correlation between dietary potassium intake and BP [44,45]. Even the dietary sodium/potassium ratio is more closely associated with BP than either sodium or potassium alone [45]. Dietary potassium intake appears to cause natriuresis and prevent retention of sodium and thus lower BP [46]. Other physiological mechanisms underlying the blood pressure lowering effect of potassium supplementation are as follows: endothelial vascular cells and macrophages inhibit the formation of free radicals by inhibiting the proliferation of the smooth vascular muscle cells, and reducing the vascular resistance [46].

Our findings are consistent with two previous meta-analyses [6,8], but inconsistent with a third one [7]. Whelton et al [6] conducted a meta-analysis in 1997 and included 33 RCTs performed before 1995 investigating the effect of potassium supplementation on BP. They demonstrated that potassium supplementation was correlated with a remarkable reduction in the mean (95% CI) SBP and DBP of -3.11 (-1.91 to -4.31) and -1.97 (-0.52 to -3.42) mmHg, respectively. The authors recommended potassium intake for prevention and treatment of hypertension. Aburto et al [8] performed a meta-analysis in 2013, including 22 RCTs and 11 cohort studies addressing the effects of potassium supplementation on blood pressure, renal function, blood lipids, catecholamine concentrations. They reported that an increase in potassium intake could reduce SBP by 3.49 (95% CI: 1.82 to 5.15) and DBP by 1.96 (0.86 to 3.06) mmHg in adults with raised blood pressure with no important side effect on blood lipid and catecholamine concentrations or renal function. The authors suggested high dietary potassium intakes to prevent and control hypertension and stroke. Dickinson et al [7] conducted a Cochrane meta-analysis in 2006, including six RCTs comparing the effect of oral potassium supplements with placebo on primary hypertension. They reported no significant reductions in SBP (MD: -11.2, 95% CI: -25.2 to 2.7) and DBP (MD: -5.0, 95% CI: -12.5 to 2.4). They concluded that potassium supplementation has no significant effect on blood pressure. However, due to small number of RCTs, included in this meta-analysis, they suggested further investigation should be done on the basis of high quality RCTs of longer duration.

This review had some limitations as follows. We planned to perform change score analysis rather than comparing post-treatment mean BP. However, only 8 out of 23 trials reported the baseline SPB and DBP. Therefore, we performed change-score analysis just based on eight trials. The review did not include studies enrolling individuals with normal BP. Thus, the results of this review should not be interpreted to include normotensive people. We could not differentiate the effect of various types of potassium provided in supplements because all studies used potassium chloride except one that used potassium aspartate [21]. Finally, we could not assess the possibility of difference in BP by gender, because 21 out of 23 RCTs were in mixed populations of males and females.

## Conclusion

This meta-analysis provided evidence based on RCTs of the effect of potassium intake on BP in hypertensive patients. Our findings indicated that potassium supplementation is a safe medication with no important adverse effects that has a modest but significant impact BP and may be recommended as an adjuvant antihypertensive agent for patients with essential hypertension.

## Supporting information

S1 PRISMA Checklist (DOC)
